# Loneliness Mediates the Relationship Between Early Life Stress and Perceived Stress but not Hypothalamic–Pituitary–Adrenal Axis Functioning

**DOI:** 10.3389/fpsyg.2021.647265

**Published:** 2021-09-03

**Authors:** Isabel Crespo-Sanmiguel, Mariola Zapater-Fajarí, Matias M. Pulopulos, Vanesa Hidalgo, Alicia Salvador

**Affiliations:** ^1^Laboratory of Social Cognitive Neuroscience, Psychobiology-IDOCAL, University of Valencia, Valencia, Spain; ^2^Department of Psychology and Sociology, Area of Psychobiology, IIS Aragón, University of Zaragoza, Teruel, Spain

**Keywords:** early life stress, loneliness, perceived stress, hypothalamic–pituitary–adrenal axis, adulthood

## Abstract

Many authors have proposed that early life stress (ELS) provokes a dysregulation of the hypothalamic–pituitary–adrenal (HPA) axis and contributes negatively to the management of stress in adulthood. However, these associations have not always been observed, making it necessary to include new factors that could explain the different results found. In this regard, people with ELS experiences report less social support during adulthood, suggesting that loneliness could be a mediating factor. Thus, our aims were to investigate whether ELS was related to both perceived stress and diurnal HPA axis activity, and whether loneliness mediates these relationships, in a community sample (*N*=187, 18–55years old). Fourteen cortisol samples were collected on two non-consecutive days to obtain the overall diurnal cortisol, diurnal cortisol slope, and bedtime levels. Additionally, ELS was assessed with the Risky Families Questionnaire (RFQ) and the Recalled Childhood and Adolescence Perceived Stress (ReCAPS) measure. Results revealed that ELS was associated with perceived stress, but not HPA axis functioning, and loneliness mediated the relationship between ELS and perceived stress, but not between ELS and HPA axis functioning. Similar results were found for both ELS questionnaires, suggesting that the ReCAPS is an adequate tool. These results highlight the importance of loneliness in understanding the long-term effects of ELS, and they indicate different effects of ELS on subjective and physiological stress indicators.

## Introduction

Early life stress (ELS) is usually operationalized as a wide variety of adverse experiences that occur in the first stages of the individual’s development, and they include negligence, socioeconomic disadvantage, physical or psychological maltreatment, or early parental loss, among others (Fogelman and Canli, 2018). Although this exposure to stress takes place during childhood and/or adolescence, the relational, emotional, and neurobiological consequences may persist throughout life (Kessler et al., 2010; Nemeroff, 2016; Lähdepuro et al., 2019). In fact, as the stress-sensitization model proposes, the ELS-related negative effects may enhance vulnerability to several stress-related psychopathological conditions (McLaughlin et al., 2010), such as posttraumatic stress disorder (Kiser et al., 1991; Yehuda et al., 2010), anxiety (Heim and Nemeroff, 2001; Lähdepuro et al., 2019), depression (Colman and Ataullahjan, 2010; Gallo et al., 2017), eating disorders (Su et al., 2016), psychosis (Read et al., 2005), bipolar disorder (Post et al., 2015), and substance abuse (Scheller-Gilkey et al., 2004; Keyes et al., 2012).

This predisposition could be due to the fact that having experienced trauma during childhood or adolescence increases vulnerability to the effects of subsequent stressful events, which aggravates the health consequences of stressors in adulthood (Hammen et al., 2000; McLaughlin et al., 2010). That is, ELS influences the capability to manage stress in adulthood, which would act as an important factor related to health. In this context, studies have shown that adults who experienced overall childhood maltreatment express more difficulties when facing a stressful episode (Han et al., 2016), which could be explained by poor management in the response to stress, as in the use of less adaptive coping strategies (Hyman et al., 2007). Likewise, it has been observed that having suffered from overall early maltreatment is associated with a greater perception of stress during adulthood in different types of populations, such as individuals with cocaine dependence in periods of abstinence (Hyman et al., 2007), female inmates (Brewer-Smyth and Burgess, 2008), and breast cancer (Han et al., 2016) or coronary artery (Bossé et al., 2018) patients, although fewer studies have been carried out in the general population (Betz et al., 2020).

Moreover, ELS has been investigated in relation to the physiological stress system via hypothalamic–pituitary–adrenal (HPA) axis functioning (Baes et al., 2014; Schalinski et al., 2015; Juruena et al., 2020), with a wide variety of results reported (Fogelman and Canli, 2018). In healthy individuals, the daily cortisol rhythm is characterized by a marked increase the first 30–45min after awakening, followed by a constant decrease until nighttime (Clow et al., 2004, 2010; Elder et al., 2014). An HPA axis dysregulation, reflected in higher overall diurnal cortisol secretion and a flattened diurnal cortisol slope (DCS), has been related to different health outcomes (Miller et al., 2007; Adam et al., 2017). Although many studies have investigated the association between ELS and the cortisol awakening response (CAR; Meinlschmidt and Heim, 2005; Heim et al., 2009; Wielaard et al., 2018) or its relationship with baseline stress and reactivity to a psychosocial stressor (Flory et al., 2009; Andreotti et al., 2015; Cărnuţă et al., 2015; Janusek et al., 2017), little is known about ELS in relation to overall diurnal cortisol, DCS, and bedtime cortisol levels.

Specifically, in the relationship between these three cortisol indexes and ELS, studies that include the period of childhood and adolescence have mostly found mixed results, depending on the cortisol index employed and the type of ELS. Thus, considering overall diurnal cortisol, men who have experienced parental loss presented higher values on this index than men with temporary parental separation (Nicolson, 2004). Although childhood economic and social adversities are not related to overall diurnal cortisol in the general population (Karlamangla et al., 2019), in a prospective study with male twins, a positive relationship was found (Franz et al., 2013). Furthermore, no relationship was found between physical and emotional abuse and neglect and overall diurnal cortisol in healthy women (Klaassens et al., 2009) or between parental bipolar disorder and overall diurnal and bedtime cortisol levels (Schreuder et al., 2016). Regarding DCS, a prospective study reported that adoptees who experienced severe neglect before the adoption presented a flatter DCS, whereas adoptees who experienced severe abuse presented a steeper DCS (Van der Vegt et al., 2009). Childhood sexual abuse has been associated with flatter DCS in female prison inmates (Brewer-Smyth and Burgess, 2008) and in women with chronic pain (Nicolson et al., 2010); however, no associations between sexual or other types of abuse and the DCS were found in pregnant women (Bublitz and Stroud, 2012). Moreover, Franz et al. (2013) showed that childhood disadvantage does not affect the DCS in males. In this context, it is important to further investigate the relationships and factors that might explain the association between ELS and stress indicators during adulthood.

It is also worth noting that ELS is a risk factor that can affect the individual’s social functioning, given that individuals who have experienced ELS report less social support (Germine et al., 2015; Beutel et al., 2017) or fewer benefits of this support, perhaps because ELS experiences negatively affect the ability to be interested in and conserve interpersonal affective ties, leading to unsatisfactory social relationships (Repetti et al., 2002). This implies a lack of companionship, emotional and instrumental support, and expressions of positive affect by others (Barrera, 1986), all of which translate into greater feelings of loneliness.

Loneliness is a complex concept that involves the subjective and painful experience of perceiving a deficient quantity and quality of desired social relationships (Peplau and Perlman, 1982). Loneliness has been associated with high perceived stress and several symptoms related to it, such as sleep disorders or chronic interpersonal stress (Yaacob et al., 2009; Doane and Adam, 2010; Yarcheski et al., 2011; Doane and Thurston, 2014; Matthews et al., 2019). Moreover, previous research observed a relationship between loneliness and HPA axis functioning, specifically, higher diurnal cortisol levels (Lai et al., 2018, 2019; Campagne, 2019), a flattened DCS (Doane and Adam, 2010; Johar et al., 2020), and a steeper DCS (Lai et al., 2019). Therefore, loneliness could be a factor that plays an important role in the relationship between ELS and both the perception of stress and HPA axis functioning in adulthood.

Based on the above, the aims of this study were to investigate the association between ELS and adult perceived stress and HPA axis functioning, and whether loneliness mediates these relationships. Specifically, we expected that higher ELS would be related to higher perceived stress. Moreover, given the heterogeneity in the results on the relationship between ELS and HPA axis functioning, and because this is the first study to investigate loneliness as a mediator between ELS and perceived stress and HPA axis indicators, our aims were to explore the existence and directionality of these relationships. Additionally, we aimed to test whether a 3-item non-standardized questionnaire on overall ELS (Recalled Childhood and Adolescence Perceived Stress, ReCAPS) is a valid tool to measure ELS. To do this, in addition to using the Risky Family Questionnaire to assess ELS, the analyses will be replicated using the ReCAPS Questionnaire.

## Materials and Methods

### Participants

The final sample in our study was composed of 187 healthy volunteers (108 men and 79 women). The data were collected by the Laboratory for the Study of Stress, Immunity, and Disease (2016) at Carnegie Mellon University under the directorship of Sheldon Cohen, PhD, and they were accessed via the Common Cold Project website (www.commoncoldproject.com; grant number NCCIH AT006694). The participants were recruited from Pennsylvania metropolitan areas through newspaper advertisements, as part of the Pittsburgh Cold Study 3 (PCS3), a prospective viral challenge study with data collected from 2007–2011. The participants’ ages ranged from 18 to 55years, with a mean of 30.39±10.98. Table 1 shows the characteristics of the study sample.

**Table 1:** Sample characteristics and descriptive statistics.

	Mean/n	SE
Age (years)	30.39	10.98
**Sex**
Female	79	
Male	108	
Educational level reached in years	14.15	1.84
**Ethnicity**
White/Caucasian	130	
Black, African-American	48	
Native American, Eskimo, Aleut	1	
Asian or Pacific Islander	3	
Hispanic, Latino	3	
Other	2	
Body mass index (kg/m^2^)	27.34	6.34
Loneliness	5.34	1.92
Perceived stress (PSS)	11.87	5.75
RFQ	27.58	10.14
ReCAPS	3.05	1.26
**Cortisol indexes**
AUCg	3.68	0.20
DCS	−0.04	1.83
Bedtime levels	0.39	0.40

RFQ, Risky family Questionnaire; ReCAPS, Recalled Childhood and Adolescence Perceived Stress; AUCg, area under the curve with respect to ground; DCS, Diurnal cortisol slope; and SE, standard error.

Two hundred and thirteen participants were recruited for the entire research project. Of the exclusion criteria for participating in the entire study protocol (www.commoncoldproject.com), for the present study, we considered the following: females who were currently lactating (breast-feeding) or pregnant; people who were currently taking sleeping pills, tranquilizers, steroids, immunosuppressants, or other regular medication regimens; individuals diagnosed with a psychiatric disorder treated within the past year or psychiatric hospitalization within the past 5years; and individuals with a history of a cardiovascular (heart) disorder, diabetes, or another chronic illness. These exclusion criteria, along with demographic and clinical data information, were evaluated in an interview held in the Children’s Hospital of Pittsburgh in a first screening session. Of the 213 participants, 26 were excluded from the data analyses in the current study because their cortisol indexes could not be calculated due to missing data.

### Procedure

Participants provided informed consent and received $1,000 for their participation in the whole protocol. The study was approved by the Carnegie Mellon University and University of Pittsburgh institutional review boards. The protocol for the whole project lasted between 14 and 16weeks, and 10 to 12weeks after the beginning of the study, the participants were infected with a virus to investigate susceptibility to the common cold. In the current study, we focus on the data available for the assessment of ELS, diurnal cortisol levels, current perceived stress, loneliness, and sociodemographic information. Although the objective of PCS3 was to observe the effect of a virus inoculation, this does not affect the psychosocial variables we investigated in the current study because they were evaluated during the visits to the hospital before the inoculation, using the self-reported questionnaires detailed below. The whole protocol is described in detail at the Common Cold Project website (www.commoncoldproject.com). Below we present the factors considered in the current study.

### Measures

#### Early Life Stress

The ELS was evaluated by two questionnaires: Risky Family Questionnaire (RFQ) and ReCAPS.

#### Risky Family Questionnaire

This questionnaire refers to adverse environmental, physical, emotional, and mental abuse or a neglectful home, among others. It was adapted (Taylor et al., 2004) from an instrument originally created to evaluate the association between family stress and health outcomes in adulthood (Felitti et al., 1998). It was composed of 13 items rated on a 5-point Likert scale (from 1 = not at all to 5 = very often). Examples of items are as: (1) “How often did a parent or other adult in the household push, grab, shove, or slap you?”; (2) “Would you say you were neglected while you were growing up, left on your own to fend for yourself?”; and (3) “In your childhood, did you live with anyone who was a problem drinker or alcoholic, or who used street drugs?”. Internal consistency of this scale for the study sample had a Cronbach’s α=0.90.

#### Recalled Childhood and Adolescence Perceived Stress

To assess overall ELS, participants completed the ReCAPS scale, which was created by the experimenters for the original study (PCS3). Participants were asked to rate their level of overall stress compared to other people with similar ages, using the same item three times: “For this age, indicate your level of overall stress compared to other people your age,” with reference to the ages of 5, 10, and 15years old. The participants had to answer using a 6-point Likert rating scale (from 1 = much less stress to 6 = much more stress). The outcome used was the mean of the three periods evaluated. Internal consistency of this scale for the study sample had a Cronbach’s α=0.78.

#### Loneliness

Loneliness was measured using the Short Loneliness Scale (Hughes et al., 2004). This scale has three items rated on a 4-point Likert scale (from 1 = never to 4 = very often). These items are as: (1) “In general, how often do you feel that you lack companionship?”; (2) “In general, how often do you feel left out?”; and (3) “In general, how often do you feel isolated from others?”. Internal consistency of this scale had a Cronbach’s α=0.80 for the study sample.

#### Perceived Stress

The degree to which people perceived their lives as stressful, uncontrollable, unpredictable, and overloaded was measured using the 10-item perceived stress scale (PSS; Cohen et al., 1983; Cohen and Janicki-Deverts, 2012). The respondents had to answer using a 5-point Likert scale (from 0 = never to 4 = very often). Sample items were as: “In the last month, how often have you felt you were unable to control the important things in your life?” and “In the last month, how often have you felt nervous and ‘stressed’?”. Internal consistency of this scale had a Cronbach’s α=0.70 for the study sample.

### Cortisol Measurements

Fourteen salivary samples were collected to assess participants’ cortisol levels using Salivettes (Sarstedt, Rommelsdorf, Germany). The saliva samples were collected 1, 2, 4, 7, 9, 11, and 14h after awakening on two non-consecutive days in their natural environment and while carrying out their usual daily activities. Participants were told to place the cotton roll in their mouth, chew on it until it became saturated, place it in the inner vial of the Salivette, and then tightly cap the outer tube. They were instructed not to eat, smoke, or brush their teeth during the 30min before the collection. Volunteers were taught and given written instructions about how and when to perform the saliva samples and the number of Salivettes. Additionally, they received a pre-programmed handheld device that identified each sample and provided a unique alphanumeric code for each. Subjects recorded this code and added the exact date and time of the samples. Moreover, they were given saliva collection records to complete after collecting the last sample on each evaluation day. They were instructed to seal and store their samples in the refrigerator until they brought them to the researchers on the baseline day of the quarantine for virus inoculation. These storage conditions ensure the stability of saliva cortisol concentrations (Garde and Hansen, 2005; Nalla et al., 2015). Cortisol levels were processed by the laboratory of Dr. Clemens Kirschbaum in Dresden (Germany), and they were determined by using time-resolved fluorescence immunoassay with a cortisol-biotin conjugate as a tracer (Dressendörfer et al., 1992). Intra- and inter-assay variabilities were each less than 12%. For each cortisol sample, there was a time window within which the samples were collected. These windows were between 45min and 90min after waking for the first sample, and between one hour before and after the established collection time for the rest of the samples. As a control measure, and to homogenize the cortisol concentrations, all the samples used were collected within these time windows, and participants who collected saliva samples outside these time windows were not included in this study. Saliva samples were adjusted according to the waking time to control for differences in cortisol levels due to variations in the awakening time. We computed three indexes, AUCg, DCS, and bedtime levels, using the average of the saliva samples on the 2days. If the participant only had the samples from 1day (*N*= 8 for AUCg, *N*=38 for DCS, and *N*=24 for bedtime cortisol), we only used the cortisol value that could be obtained. The AUCg reflects the overall diurnal cortisol secretion. AUCg was computed using all the saliva samples, and it was calculated using the trapezoidal formula proposed by Pruessner et al. (2003). The DCS reflects the decrease in cortisol levels during the day, and it was calculated by regressing the cortisol values from the second sample to the last sample for each participant. In the case of the DCS, a larger value indicates a flatter slope (less cortisol decline throughout the day), whereas a smaller value indicates a steeper slope (greater diurnal decline). Finally, bedtime cortisol was calculated as the mean cortisol before going to sleep on the 2days.

### Statistical Analyses

**Figure 1:**
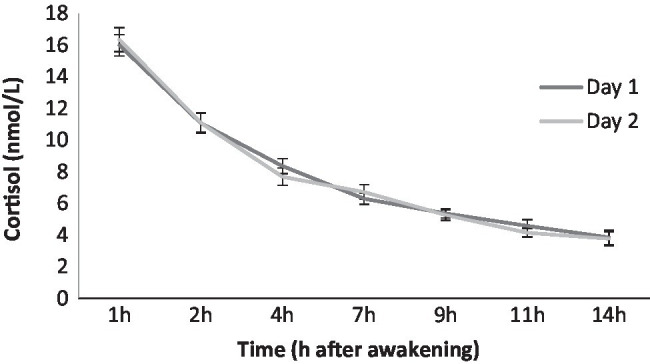
Diurnal cortisol profile of each day (raw data).

Figure 1 shows the diurnal cortisol profile of each day using raw data. First, because the cortisol levels did not follow a normal distribution, they were log transformed. To verify that the cortisol data really reflected the baseline functioning of the HPA axis, correlation analyses were performed between the two days at each collection time, which allowed us to use the average cortisol levels (*p*≤0.047). To determine the covariates that would be included in the regression and mediation analyses, Pearson’s correlations were performed between all the variables included in the current study. Only the sociodemographic variables that were significantly related to the main factors of the study (i.e., ELS, loneliness, perceived stress, and cortisol indexes) were included as covariates (see Table 2). Thus, sex was associated with the three cortisol indexes, with men having higher cortisol levels. Therefore, sex was a covariate in all the regression and mediation analyses that included cortisol indexes. Years of education was positively associated with the ReCAPS, and so it was included as a covariate in all the regression and mediation analyses that included the ReCAPS.

**Table 2:** Pearson’s correlations between all variables studied.

	Sex	Education	Ethnicity	BMI	Loneliness	PSS	RFQ	ReCAPS	AUCg	DCS	Bedtime
Age	0.060	−0.023	0.153^*^	0.312^**^	0.130	−0.102	−0.144	0.034	0.063	0.102	0.143
Sex		0.089	0.139	0.176^*^	0.030	−0.032	0.016	0.133	−0.166^*^	−0.233^**^	−0.182^*^
Education			−0.269^**^	−0.233^**^	0.054	0.067	−0.055	0.237^**^	−0.059	−0.065	−0.043
Ethnicity				0.275^*^	0.005	0.005	0.101	−0.105	−0.024	0.041	0.082
BMI					−0.015	0.005	0.075	−0.044	−0.030	0.019	0.016
Loneliness						0.430^**^	0.299^**^	0.333^**^	−0.064	0.040	−0.007
PSS							0.201^**^	0.193^*^	−0.029	−0.016	−0.049
RFQ								0.438^**^	−0.025	−0.009	0.003
ReCAPS									−0.075	−0.054	−0.084
AUCg										0.738^**^	0.487^**^
DCS											0.549^**^

BMI, body mass index; PSS, perceived stress scale; RFQ, Risky family Questionnaire; ReCAPS, Recalled Childhood and Adolescence Perceived Stress; AUCg, area under the curve with respect to ground; and DCS, Diurnal cortisol slope.

* *p*<0.05 level.

** *p*<0.01 level (two tailed).

We used PROCESS 3.4 for SPSS to test mediation effects. It makes possible to estimate the indirect effect of ELS on PSS and on the cortisol indexes (AUCg, DCS, and bedtime levels) via loneliness, which is equivalent to the difference between the total effect (relationship between ELS and PSS/cortisol indexes, not controlling for loneliness) and the direct effect of the independent factor (relationship between ELS and PSS/cortisol indexes, controlling for loneliness). To determine the significance of the indirect effect, PROCESS uses bootstrapped bias-corrected 95% confidence intervals (Hayes, 2017) of the interaction effect with 5,000 bootstrapped samples. We interpret that there is a significant indirect effect when this confidence interval does not contain zero. The mediation analyses were performed including the covariates.

Post-hoc statistical power analyses were performed with de G^*^Power program, estimating a power >0.80 with an alpha level *p*=0.05 and an *N*=187 for regression analyses between ELS and PSS and cortisol indexes. Only the relationship between ELS and AUCg cortisol index has statistical power of 0.70. The mediation analyses use bootstrapping technique that draws random sample of a fixed sample size with replacement from the dataset, which increases the statistical power. The sample size is considered and this statistical approach controls for this factor in the analyses (Hayes, 2017).

Tolerance values indicate that there are no collinearity issues for the factors included in the model (i.e., tolerance >0.01). In this study, we used the multivariate outliers, and the number of outliers for each regression and mediation analysis is indicated for each analysis in Tables 3, 4, and 5, respectively. We considered the values outliers when they differed by more than±3 SD and, thus, were eliminated from the regression and mediation analyses. Statistical analyses were carried out using SPSS v.24 (IBMS Statistics, Chicago, IL, United States). All *p* values were two tailed, and the level of significance was taken as *p<*0.05.

**Table 3:** Adjusted regression analyses with RFQ and ReCAPS as predictors and PSS and cortisol indexes as dependent variable.

		PSS	AUCg	DCS	Bedtime
RFQ	Change R^2^	0.041	0.001	0.000	0.000
	Adj. R^2^	0.036	0.021	0.058	0.032
	Beta (standardized)	0.203	−0.023	0.014	−0.017
	*p*	0.006	0.749	0.840	0.814
	Outliers	1	2	3	1
ReCAPS	Change R^2^	0.033	0.003	0.000	0.004
	Adj. R^2^	0.027	0.024	0.058	0.036
	Beta (standardized)	0.181	−0.059	0.018	−0.067
	*p*	0.014	0.423	0.809	0.356
	Outliers	1	2	3	1

The analyses were controlled by educational level for the analyses that include ReCAPS and by sex for the analyses that include the cortisol indexes. RFQ, Risky Family Questionnaire; ReCAPS, Recalled Childhood and Adolescence Perceived Stress; PSS, perceived stress scale; AUCg, area under the curve with respect to ground; and DCS, Diurnal cortisol slope.

**Table 4:** Adjusted mediation models of the relationship between ELS (measured by RFQ) as predictor and perceived stress and cortisol levels (AUCg, DCS, and bedtime) as dependent variables via loneliness.

Dependent variable: PSS (1 outlier)						
	Effect	SE	*t*	*p*	LLCI	ULCI
RFQ to loneliness	0.299	0.070	4.245	<0.001	0.160	0.437
Loneliness to PSS	0.401	0.067	5.951	<0.001	0.268	0.534
Indirect effect	0.120	0.042	-	-	0.048	0.209
Total effect	0.196	0.070	2.805	0.006	0.058	0.335
Direct effect	0.077	0.067	1.140	0.256	−0.056	0.210
**Dependent variable: AUCg (2 outliers)**						
	**Effect**	**SE**	***t***	***p***	**LLCI**	**ULCI**
RFQ to loneliness	0.299	0.071	4.221	<0.001	0.159	0.439
Loneliness to AUCg	−0.038	0.072	−0.523	0.601	−0.180	0.104
Indirect effect	−0.011	0.022	-	-	−0.058	0.028
Total effect	−0.022	0.069	−0.320	0.749	−0.158	0.114
Direct effect	−0.011	0.072	−0.149	0.882	−0.153	0.132
**Dependent variable: DCS (3 outliers)**						
	**Effect**	**SE**	***t***	***p***	**LLCI**	**ULCI**
RFQ to loneliness	0.296	0.071	4.171	<0.001	0.156	0.436
Loneliness to DCS	0.101	0.113	0.887	0.376	−0.123	0.324
Indirect effect	0.030	0.031	-	-	−0.027	0.096
Total effect	0.022	0.108	0.202	0.840	−0.192	0.235
Direct effect	−0.008	0.113	−0.070	0.944	−0.232	0.216
**Dependent variable: Bedtime levels (1 outlier)**						
	**Effect**	**SE**	***t***	***p***	**LLCI**	**ULCI**
RFQ to loneliness	0.293	0.071	4.153	<0.001	0.154	0.433
Loneliness to bedtime	−0.022	0.074	−0.302	0.763	−0.168	0.123
Indirect effect	−0.007	0.019	-	-	−0.043	0.032
Total effect	−0.017	0.070	−0.236	0.814	−0.155	0.122
Direct effect	−0.010	0.074	−0.137	0.892	−0.155	0.135

The analyses were controlled by sex for the analyses that include the cortisol indexes. PSS, perceived stress scale; RFQ, Risky Family Questionnaire; AUCg, area under the curve with respect to ground; DCS, Diurnal cortisol slope; and SE, Standard error.

**Table 5:** Adjusted mediation models of the relationship between ELS (measured by ReCAPS) as predictor and perceived stress and cortisol levels (AUCg, DCS, and bedtime) as dependent variables via loneliness.

Dependent variable: PSS (1 outlier)						
	Effect	SE	*t*	*p*	LLCI	ULCI
ReCAPS to loneliness	0.339	0.072	4.720	<0.001	0.197	0.481
Loneliness to PSS	0.411	0.069	6.003	<0.001	0.276	0.547
Indirect effect	0.140	0.040	-	-	0.068	0.225
Total effect	0.174	0.073	2.389	0.018	0.030	0.317
Direct effect	0.034	0.071	0.485	0.629	−0.105	0.173
**Dependent variable: AUCg (2 outliers)**						
	**Effect**	**SE**	***t***	***p***	**LLCI**	**ULCI**
ReCAPS to loneliness	0.343	0.073	4.711	<0.001	0.199	0.486
Loneliness to AUCg	−0.026	0.073	−0.357	0.721	−0.170	0.118
Indirect effect	−0.009	0.025	-	-	−0.063	0.035
Total effect	−0.050	0.071	−0.704	0.482	−0.191	0.090
Direct effect	−0.041	0.076	−0.545	0.587	−0.191	0.108
**Dependent variable: DCS (3 outliers)**						
	**Effect**	**SE**	***t***	***p***	**LLCI**	**ULCI**
ReCAPS to loneliness	0.339	0.073	4.661	<0.001	0.196	0.483
Loneliness to DCS	0.101	0.115	0.879	0.380	−0.126	0.328
Indirect effect	0.034	0.038	-	-	−0.042	0.111
Total effect	0.022	0.112	0.193	0.847	−0.200	0.243
Direct effect	−0.013	0.119	−0.107	0.915	−0.248	0.222
**Dependent variable: Bedtime levels (1 outlier)**						
	**Effect**	**SE**	***t***	***p***	**LLCI**	**ULCI**
ReCAPS to loneliness	0.339	0.072	4.695	<0.001	0.196	0.481
Loneliness to bedtime	−0.004	0.075	−0.056	0.956	−0.152	0.143
Indirect effect	−0.001	0.020	-	-	−0.043	0.037
Total effect	−0.065	0.073	−0.892	0.374	−0.208	0.079
Direct effect	−0.063	0.077	−0.822	0.412	−0.215	0.089

All analyses were controlled by educational level and for the analyses that include the cortisol indexes, in addition to educational level, sex. PSS, perceived stress scale; ReCAPS, Recalled Childhood and Adolescence Perceived Stress; AUCg, area under the curve with respect to ground; DCS, Diurnal cortisol slope; and SE, Standard error.

Additionally, we performed moderated regression analyses between ELS (i.e., RFQ and ReCAPS) and perceived stress and the three cortisol indexes with sex as a moderating factor to explore possible sex differences. The sex factor did not moderate any association (all *p*>0.158).

## Results

### Correlation Analyses

The Pearson’s correlation between the RFQ and ReCAPS showed that the two questionnaires were positively and strongly related [*r* (185)=0.438, *p*<0.001]. After controlling for years of education, due to its positive relationship with ReCAPS, the partial correlation showed the same statistical result [*r* (184)=0.465, *p*<0.001].

Table 2 shows Pearson’s correlations between all the variables used in the study. Results showed that the PSS was positively related to the RFQ [*r* (185)=0.201, *p*=0.006] and ReCAPS [*r* (185)=0.193, *p*=0.008]. However, RFQ was not related to any cortisol index [AUCg: *r* (185)=−0.025, *p*=0.735; DCS: *r* (185)=−0.009, *p*=0.902; Bedtime levels: *r* (185)=0.003, *p*=0.971] and neither ReCAPS [AUCg: *r* (185)=−0.075, *p*=0.311; DCS: *r* (185)=−0.054, *p*=0.460; Bedtime levels: *r* (185)=−0.084, *p*=0.250]. In addition, loneliness was positively related to RFQ [*r* (185)=0.299, *p*<0.001], ReCAPS [*r* (185)=0.333, *p*<0.001], and PSS [*r* (185)=0.430, *p*<0.001], but not to the cortisol outputs [AUCg: *r* (185)=−0.064, *p*=0.383; DCS: *r* (185)=0.040, *p*=0.586; Bedtime levels: *r* (185)=−0.007, *p*=0.921].

### Regression Analyses

Regression analyses showed that the RFQ was positively associated with the PSS (*B*=0.203, *p*=0.006), but not with any cortisol indexes (all *p*>0.749). When the regression analyses were re-analyzed using ReCAPS as an indicator of ELS, results showed similar significance. That is, ReCAPS was positively associated with PSS (*B*=0.181, *p*=0.014), but no associations were found between ReCAPS and any cortisol indexes (all *p*>0.356; Table 3).

### Mediation Analyses Between ELS and Perceived Stress *via* Loneliness

Mediation analyses revealed that higher RFQ scores were associated with greater loneliness (*B*=0.299, IC 95% [0.160, 0.437]). Moreover, people who showed greater loneliness had higher scores on the PSS (*B*=0.401, IC 95% [0.268, 0.534]). The indirect effect (i.e., effect of RFQ on PSS via loneliness) was statistically significant (*B*=0.120, IC 95% [0.048, 0.209]). In addition, the total effect (i.e., effect of RFQ on PSS, without considering loneliness) was significant (*B*=0.196, IC 95% [0.058, 0.335]). However, the direct effect (i.e., effect of RFQ on PSS, controlling for loneliness) was not significant (*B*=0.077, IC 95% [−0.056, 0.210]; Table 4).

Results of the mediation analyses using ReCAPS showed that higher ReCAPS scores were associated with greater loneliness (*B*=0.339, IC 95% [0.197, 0.481]). Moreover, people who showed greater loneliness had higher scores on the PSS (*B*=0.411, IC 95% [0.276, 0.547]). The indirect effect (i.e., effect of ReCAPS on PSS via loneliness) was statistically significant (*B*=0.140, IC 95% [0.068, 0.225]). In addition, the total effect (i.e., effect of ReCAPS on PSS, without considering loneliness) was significant (*B*=0.174, IC 95% [0.030, 0.317]). However, the direct effect (i.e., effect of ReCAPS on PSS, controlling for loneliness) was not significant (*B*=0.034, IC 95% [−0.105, 0.173]; Table 5).

### Mediation Analyses Between ELS and Cortisol *via* Loneliness

Mediation analyses revealed that higher RFQ scores were associated with greater loneliness in the analyses with the three cortisol indexes (for AUCg: *B*=0.299, IC 95% [0.159, 0.439], for DCS: *B*=0.296, IC 95% [0.156, 0.436], and for bedtime: *B*=0.293, IC 95% [0.154, 0.433]). However, people who showed more loneliness did not show significant results on any cortisol index (for AUCg: *B*=−0.038, IC 95% [−0.180, 0.104], for DCS: *B*=0.101, IC 95% [−0.123, 0.324], and for bedtime: *B*=−0.022, IC 95% [−0.168, 0.123]). The indirect effects (i.e., effect of RFQ on cortisol levels via loneliness) were not significant (for AUCg: *B*=−0.011, IC 95% [−0.058, 0.028] for DCS: *B* =0.030, IC 95% [−0.027, 0.096], and for bedtime: *B*=−0.007, IC 95% [−0.043, 0.032]). Neither the total effect (i.e., effect of RFQ on cortisol levels, without considering loneliness; for AUCg: *B*=−0.022, IC 95% [−0.158, 0.114] for DCS: *B* =0.022, IC 95% [−0.192, 0.235], and for bedtime: *B*=−0.017, IC 95% [−0.155, 0.122]) nor the direct effects (i.e., effect of RFQ on cortisol levels, controlling for loneliness; for AUCg: *B*=−0.011, IC 95% [−0.153, 0.132] for DCS: *B*=−0.008, IC 95% [−0.232, 0.216], and for bedtime: *B*=−0.010, IC 95% [−0.155, 0.135]) were significant either (Table 4).

Mediation analyses using ReCAPS showed that higher ReCAPS scores were associated with greater loneliness in the analyses with the three cortisol indexes (for AUCg: *B* =0.343, IC 95% [0.199, 0.486], for DCS: *B* =0.339, IC 95% [0.196, 0.483], and for bedtime: *B* =0.339, IC 95% [0.196, 0.481]). However, people who showed more loneliness did not show significant results on any cortisol index (for AUCg: *B*=−0.026, IC 95% [−0.170, 0.118], for DCS: *B* =0.101, IC 95% [−0.126, 0.328], and for bedtime: *B*=−0.004, IC 95% [−0.152, 0.143]). The indirect effects (i.e., effect of ReCAPS on cortisol levels via loneliness) were not significant (for AUCg: *B*=−0.009, IC 95% [−0.063, 0.035], for DCS: *B*=0.034, IC 95% [−0.042, 0.111], and for bedtime: *B*=−0.001, IC 95% [−0.043, 0.037]). Neither the total effects (i.e., effect of ReCAPS on cortisol levels, without considering loneliness; for AUCg: *B*=−0.050, IC 95% [−0.191, 0.090], for DCS: *B*=0.022, IC 95% [−0.200, 0.243], and for bedtime: *B*=−0.065, IC 95% [−0.208, 0.079]) nor the direct effects (i.e., effect of ReCAPS on cortisol levels, controlling for loneliness; for AUCg: *B*=−0.041, IC 95% [−0.191, 0.108], for DCS: *B*=−0.013, IC 95% [−0.248, 0.222], and for bedtime: *B*=−0.063, IC 95% [−0.215, 0.089]) were significant either (Table 5).

## Discussion

The aims of this study were to investigate whether ELS was associated with the current perception of stress and the HPA axis functioning in adulthood, and the mediating role of loneliness in these relationships. In addition, we wanted to check whether the ReCAPS is an adequate and useful tool to measure overall ELS. The results showed that ELS was related to perceived stress, but not to HPA axis functioning. Moreover, loneliness mediated the relationship between ELS and perceived stress, but not the relationship with the HPA axis. Additionally, RFQ and ReCAPS were strongly associated, and similar results were found with both questionnaires.

Regarding the relationship between ELS and perceived stress, the results indicated that ELS, evaluated as emotional and physical abuse and neglect, was associated with a higher perception of stress in adulthood. Despite the different types of samples studied, this result agrees with previous studies that reported significant relationships between childhood maltreatment (assessed by the Childhood Trauma Questionnaire; CTQ) and perceptions of stress during adulthood (Hyman et al., 2007; Han et al., 2016; Bossé et al., 2018; Betz et al., 2020). Research has shown that a history of ELS can influence the way stress is perceived and managed in adulthood because it can lead to vulnerability and low tolerance to stressors in later life (Hammen et al., 2000), as well as poor adaptive stress coping strategies (Hyman et al., 2007). In this regard, the relationship with attachment figures, such as parents, who are the source for learning emotional management and self-regulation in stressful situations, is important (Bowlby, 1982; Schore, 2000; Schore and Schore, 2008). However, among people who experience ELS, these relationships are neglected or disorganized, making it difficult for them to learn to self-regulate in stressful situations from an early age and into adulthood. For example, breast cancer patients who had experienced ELS had more perceived stress during the disease (Han et al., 2016). This greater perception of stress could be due to the effects of ELS on lower self-efficacy and greater helplessness as different facets of stress perception. Experiences of abandonment before the age of 18 can lead to more perceived stress and, specifically, lower perceived self-efficacy (Betz et al., 2020). In this study, we added a possible mediating factor to contribute to explaining the relationship between early and current perceived stress and HPA axis functioning, as well as the discrepant results reported by several studies.

As our results suggest, loneliness may be a mediator between ELS and the current perception of stress in adulthood. The analyses demonstrate that individuals who have experienced more ELS referred to higher scores on loneliness and, in turn, presented higher levels of perceived stress. This association between ELS and loneliness is in line with previous studies that found that individuals with ELS experiences reported less social support (Germine et al., 2015; Beutel et al., 2017), a situation that has been associated with a greater perception of loneliness (Matthews et al., 2019). This result might be explained by the fact that some adverse events, such as parental divorce or interpersonal traumas, are related to poor/inadequate representations and abilities in relationships with others (Crowell et al., 2009). These inadequate learnings are reflected in the type of interpersonal bonds established throughout life (Repetti et al., 2002; Fonagy and Luyten, 2018). In addition, our results support previous studies that report the same relationship between loneliness and perceived stress (Yaacob et al., 2009; Yarcheski et al., 2011). Two explanations can be suggested for this association. First, because the social network would act as a buffer of stressors, people who feel lonely or present poor quality social ties may suffer more from daily stressors or be more sensitive to their impact (Hawkley et al., 2008). Second, people who feel lonely tend to interpret social interactions as more threatening, due to hypersensitivity in this type of interaction. For this reason, loneliness would also be understood as a stressor in itself (Cacioppo and Hawkley, 2009).

Regarding HPA axis functioning, ELS was not directly or indirectly associated with diurnal cortisol. This finding agrees with studies that failed to find a direct relationship between ELS and overall diurnal cortisol (Klaassens et al., 2009; Schreuder et al., 2016; Karlamangla et al., 2019, respectively), DCS (Bublitz and Stroud, 2012; Franz et al., 2013), or bedtime levels (Schreuder et al., 2016), employing different questionnaires and indexes to measure ELS, such as the CTQ (Klaassens et al., 2009; Nicolson et al., 2010; Franz et al., 2013; Schreuder et al., 2016), Early Trauma Inventory (Klaassens et al., 2009), Adverse Childhood Experiences (Bublitz and Stroud, 2012), or composite indexes (Nicolson, 2004; Van der Vegt et al., 2009; Franz et al., 2013; Karlamangla et al., 2019). Although in our study an association was found between ELS and loneliness, the association between loneliness and the HPA axis was not significant, which differs from studies that found a significant association (Doane and Adam, 2010; Lai et al., 2018, 2019; Campagne, 2019; Johar et al., 2020). However, these studies did not control the potential effects of ELS. Moreover, this lack of relationship could suggest that the allostatic load approach to basal hormone levels used in our study may not be the most appropriate indicator of the effects of ELS on HPA axis functioning. Perhaps, we should focus on another type of measure, such as the dynamic range of the system given that, in individuals with ELS, lower morning cortisol peak levels or a compression of the diurnal dynamic range of cortisol have been observed (Meinlschmidt and Heim, 2005; Karlamangla et al., 2019). Thus, a range that includes the morning cortisol peak and its difference from minimum levels at rest might be more appropriate. The contradictory results can also be explained by methodological differences, such as the relatively large compliance window for cortisol collection. In addition, the ELS severity of the sample in our study was low and may be sufficient to affect the perception of stress as a long-life bias, but not severe enough to affect the HPA axis. If individuals with clinical diagnoses had been included in the study, the relationship between ELS and the HPA axis might have yielded significant results, as reported in individuals diagnosed with psychosis (Faravelli et al., 2017).

The ReCAPS questionnaire was strongly associated with the RFQ. In addition, results of the regression and mediation analyses using ReCAPS to investigate the relationship between ELS and perceived stress and HPA axis functioning and the role of loneliness in both relationships obtained the same statistical conclusions as the RFQ. These results suggest that ReCAPS could be an adequate brief tool, complementary to other ELS questionnaires, such as the RFQ used in the current study or the widely used CTQ (Bernstein and Fink, 1998). Although different types of abuse and neglect are collected in the RFQ items, the score used is an overall ELS score, without differentiating between types of stressors. A similar self-report measure, the *Global Perceived Early Life Stress Scale* (GPELS, Carpenter et al., 2004; consisting of a 6-point Likert scale, responded to in relation to what they consider normal for their peer cohort) was developed to measure ELS in adults, and it has shown sensitivity on measures of HPA axis functioning. To the best of our knowledge, longitudinal studies investigating the effect of ELS on the stress system are not comparable with our results due to the disparity in the evaluation of both ELS and cortisol (Trickett et al., 2010; Doom et al., 2014). Therefore, our results should be confirmed in future longitudinal studies. Finally, it is worth mentioning that we found a positive relationship between ReCAPS and years of education, whereas the literature on this topic reports a relationship in the opposite direction (Fors et al., 2009; Zielinski, 2009). For future research, it would be interesting to study mediating effects that explain this relationship.

In addition to the important findings, some limitations of this study must be considered. First, the cross-sectional data of the study make it impossible to reach conclusions about causal relationships. Second, both questionnaires employed to assess ELS are general and retrospective measures and, consequently, could be affected by recall bias. Prospective and longitudinal studies would greatly improve this research area, as well as the combination of general and specific measurements because there is evidence suggesting that different forms of early adversity can lead to different clinical outcomes (Bentall et al., 2012). Moreover, the recall of ELS could be affected by stressful situations during adulthood that were not explored in the current study, given that adverse events in childhood increase the risk of experiencing more stressful life events during adulthood (Shapero et al., 2014), which is associated with worse clinical results (Pearlin et al., 1981; Shevlin et al., 2008; Cotter et al., 2016; Stevens et al., 2017). However, RFQ is considered a valid instrument and has been employed in several investigations (Benedetti et al., 2011; Collazzoni et al., 2017, 2020; Counts et al., 2018; Poletti et al., 2020). Although the use of non-consecutive days for cortisol sample collection reduces the replicability of the data, the pattern of sample times used (seven samples per day) provides a large number of samples throughout the day, thus allowing a valid evaluation of diurnal HPA axis functioning. In addition, the fact that there were two collection days increases the reliability of the data (Kraemer et al., 2006).

In sum, loneliness appears to be a mediating factor between ELS and perceived stress, but not HPA axis functioning (as measured by saliva diurnal cortisol levels). Our results highlight the importance of intervening in young people who have suffered from ELS, in order to reduce the perception of loneliness and promote the quality of social network support and significant emotional ties. Loneliness interventions could also be useful to reduce the perception of stress produced by the daily stress of having fewer strategies to cope with ELS experiences and feelings of loneliness and improve the person’s state of health. These are novel findings, although more research is needed to address how loneliness mediates the association between ELS and perceived stress. In this line, unhelpful metacognitive beliefs, such as “worrying about threats means I can be prepared” or “if I continue to worry, I will lose my mind,” can arise during childhood as an attempt to manage early emotional abuse (Myers and Wells, 2015). These metacognitive beliefs may be influencing negative cognitive and emotional consequences of ELS during adulthood (Mansueto et al., 2019), which suggests that they could be an important factor to study in the relationship between ELS and feelings related to loneliness or stress. Thus, future research is needed to determine this specific pathway and others through which early adverse experiences affect psychological processes related to loneliness and stress in adulthood.

## Data Availability Statement

The datasets presented in this study can be found in online repositories. The names of the repository/repositories and accession number(s) can be found at: www.commoncoldproject.com.

## Ethics Statement

The studies involving human participants were reviewed and approved by the Carnegie Mellon University and University of Pittsburgh institutional review boards. The patients/participants provided their written informed consent to participate in this study.

## Author Contributions

IC-S, AS and VH interpreted the results, revised the literature, and wrote the manuscript. MZ-F contributed to the statistical analyses and interpreted them. MMP revised the manuscript and the statistical analyses. All authors contributed to the article and approved the submitted version.

## Conflict of Interest

The authors declare that the research was conducted in the absence of any commercial or financial relationships that could be construed as a potential conflict of interest.

## Publisher’s Note

All claims expressed in this article are solely those of the authors and do not necessarily represent those of their affiliated organizations, or those of the publisher, the editors and the reviewers. Any product that may be evaluated in this article, or claim that may be made by its manufacturer, is not guaranteed or endorsed by the publisher.
